# Incongruent virtual reality attenuates breathlessness and leg fatigue during stationary cycling

**DOI:** 10.1007/s10055-026-01391-6

**Published:** 2026-05-20

**Authors:** Lara Biller, Lucy Starling, Ayush Sinha, David Dearlove, Richard Bruce, Oliver Runswick, Stephen Taylor, Martin Sergeant, Sarah Finnegan, Kyle Pattinson

**Affiliations:** 1 Nuffield Department of Clinical Neurosciences, University of Oxford, John Radcliffe Hospital, 6th Floor, West Wing, Oxford, OX3 9DU UK; 2 Medical Sciences Division, University of Oxford, Oxford, UK; 3 Centre of Humans and Applied Physiological Sciences, King’s College London, London, UK; 4 Institute of Psychiatry, Psychology and Neuroscience, King’s College, London, UK; 5 Wellcome Centre for Human Genetics, University of Oxford, Oxford, UK

**Keywords:** Breathlessness, Perception, Effort/Exertion, Virtual Reality, Human Exercise, Bicycling, Sensation, Patient Involvement

## Abstract

This study tests whether creating incongruence between visual stimuli and pedalling resistance in virtual reality (VR) cycling can attenuate breathlessness and leg fatigue. We performed a trial in healthy participants, patient and public involvement (PPI) activities, and a feasibility study with patients with cardiac and respiratory conditions.

Healthy Participants Study: Forty-eight healthy individuals engaged in a double-blind, randomized trial comparing incongruent VR (visual slope less than pedal resistance) with a congruent control. Incongruent VR significantly reduced breathlessness and leg fatigue (effect size = 0.39, SE = 0.04, *p* < 0.001 for both). Depression and health anxiety influenced these effects, with notable associations for breathlessness (effect size = 0.29, SE = 0.138, *p* = 0.04) and leg fatigue (effect size = 0.44, SE = 0.135, *p* = 0.002).

PPI and Patient Study: Sixty-six stakeholders, including patients and caregivers, provided feedback on the paradigm’s potential through focus groups and structured interviews. A subsequent observational study with nine patients demonstrated acceptability and tolerability of the VR cycling paradigm.

The findings demonstrate that incongruent VR can attenuate breathlessness and fatigue perception, highlighting its promise for use in clinical settings, such as cardiac and pulmonary rehabilitation.

## Introduction

Diseases of the heart and lungs have a substantial impact on society. Cardiovascular Disease (CVD) and Chronic Obstructive Pulmonary Disease (COPD) account for both the second and fifth largest causes of mortality in the UK, respectively (BHF, 2022; NICE, 2018). People with these conditions often experience a poorer quality of life due to the discomfort of chronic breathlessness, a symptom which can worsen during physical exercise (Hanania and O’Donnell 2019; Johnson et al. 2017; Scano et al. 2013). The fear of becoming breathless can then lead to a ‘breathlessness spiral’, where exercise is avoided due to discomfort, thus increasing such discomfort and expediting disease progression (O’Donnell et al. 2020; Polkey and Moxham 2006). Such aversion is particularly problematic as exercise-based rehabilitation programs are key to the management of these conditions (McMahon et al. 2017). Cardiac rehabilitation, for example, is one of the most successful therapies offered by the NHS. However, rates of ~ 30% failure to complete the course of treatment remains a significant limiting factor (BHF, 2016). For many people with cardiac or respiratory conditions, these heightened sensations of breathlessness and discomfort evoked by exercise pose a considerable barrier to uptake (Almadana Pacheco et al. 2017; Resurrección et al. 2018).

Whilst such discomfort is reliant on underlying pathophysiology, a body of evidence suggests the brain may also play a vital role in mediating breathlessness perceptions (Bruce et al. 2019; Marlow et al. 2019). ‘Top-down’ processing involves the integration of incoming sensory input with (often unconscious) predictions, based on previous experience, also known as *priors*, into a Bayesian mental model (Hayen et al. 2013; Van den Bergh et al. 2017). For example, a person with COPD beginning to hyperventilate when merely looking up a staircase they have yet to climb. Indeed, a recent study found that healthy people viewing a steeper hill in virtual reality (VR), whilst cycling, experienced increased blood pressure than when viewing a less steep hill, despite no change in pedalling resistance (Bruce et al. 2025). In both instances, based upon this mental model, the relative importance of predictions within an individual’s mental model of the world were incorrectly assigned, affecting the person’s sensory experience as a result. This model of breathlessness provides opportunities for exciting new therapeutic tools, such as virtual reality (VR). VR allows for controllable simulated virtual environments that users can interact with (Kyaw et al. 2019). Because these environments can be created and customised easily, VR is already being widely used in the treatment of psychological disorders, including exposure therapy for phobia (Boeldt et al. 2019; Freeman et al. 2018). The immersive power of VR is providing a new type of personalised medicine whereby, via the manipulation of sensory input, psychological responses can be re-conditioned (Kouijzer et al. 2023; Park et al. 2019). It follows that similar principles may also be applied to perceptions of physical exertion during exercise, such as breathlessness.

Indeed, there is a growing body of research exploring the effects of exercising in VR. Studies have shown that people find exercising in VR more enjoyable and that doing so may increase the frequency of engaging in physical activity (Mouatt, 2020). Further, that immersive VR can evoke lower perceived effort than the same exercise done in a non-immersive environment (Ng, 2019). Likely, these effects are partly explained by such immersive environments providing significant distraction, the analgesic powers of which are well documented (Buhle and Wager 2010; McCaul and Malott 1984). This has already been exploited in numerous studies of pain involving VR, many showing that regular use of VR to be helpful in alleviating chronic pain and even a pilot study that found unmedicated labouring women had reduced pain scores during contractions when wearing a VR headset (Frey et al. 2019; Li et al. 2011). Another factor contributing to exercise performance differences may be VR’s power to evoke embodiment. For example, one paper found that when participants exercised in VR with a more muscular avatar, they could perform more bicep curls (Czub 2021).

Given such promising research on the power of VR to manipulate exercise performance and perceptions, it follows that these insights could be applied with certain patient populations to increase tolerance and engagement with exercise. Despite the extensive literature on VR’s distracting and immersive power, there has been less focus on exploring the effects of incongruent sensory input on perception. While virtual reality ‘exergames’ (combining exercise and gaming) exist, such as FitXR (experimentally tested by Barbour et al. 2024), none currently manipulate sensory input in the same way as our previous and current work.

Our work has utilised neuroscientific principles to create a novel VR cycling program, where participants cycle on a stationary exercise bike or ergometer along virtual hills whilst wearing a VR headset. In our first paper, we manipulated the visual slope (%) of virtual hills and, separately, the pedalling resistance (%) on the bike, so participants cycled along hills with a visual slope both congruent and incongruent to pedalling resistance (Finnegan et al. 2023). We found that manipulating visual slope significantly altered participants’ perception of breathlessness, regardless of actual pedalling resistance they cycled. These results were then supported by a follow-up pilot study, finding breathlessness and rate of perceived exertion (RPE) could be manipulated bidirectionally through incongruent VR cycling (Runswick, 2023).

The present study seeks to explore the potential of these findings further and within a therapeutic context. First, by determining if the same program can specifically attenuate feelings of breathlessness and leg fatigue by only showing visual slopes that appear less steep than those congruent to the pedalling resistance. Second, by assessing factors related to individual variability by recruiting a larger cohort of healthy volunteers. And third, by investigating if such a paradigm would be accepted by a relevant clinical population through extensive patient and participant involvement (PPI) and a small feasibility study in a group of people with cardiac and respiratory conditions.

## Study one: healthy participants

### Introduction

**Fig. 1:**
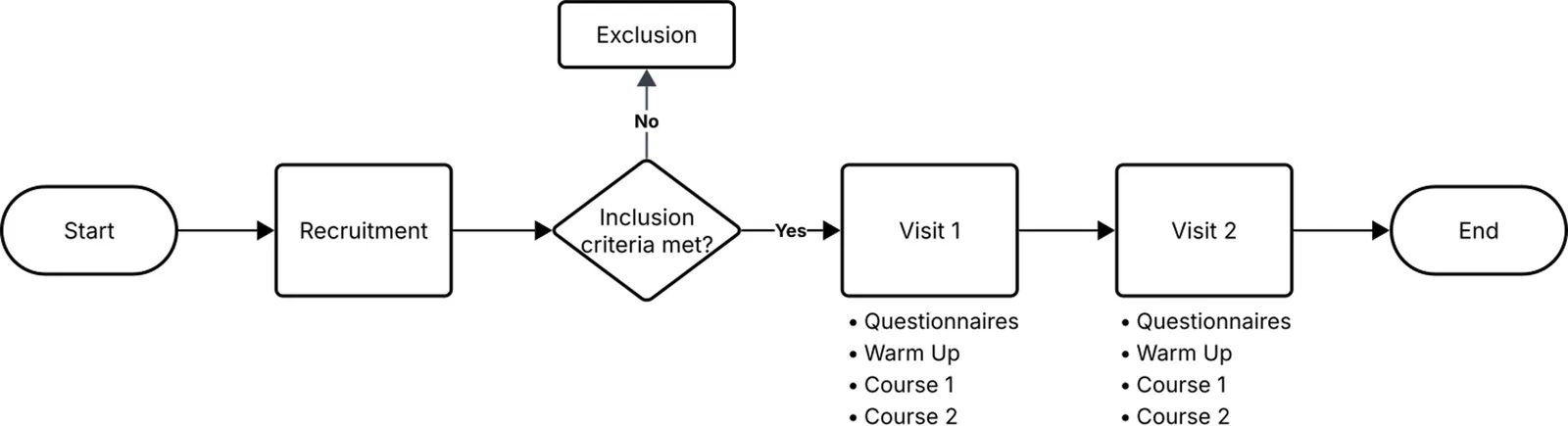
Flow chart of study one (healthy volunteers), from recruitment screenings to data collection

Our previous work demonstrated that incongruent VR stimulation could manipulate perception of breathlessness whilst cycling. The present study wishes to clarify this effect in healthy people. The aim of the study, specifically, is to investigate if, first, whether having a lower visual gradient of slope can attenuate sensations of breathlessness. Second, whether this effect can extend to feelings of leg fatigue, another sensation of exertion that provides common discomfort during exercise. Finally, whether, by recruiting a larger sample, we can get indications of interpersonal variability in response. Figure 1.

### Methods

#### Participants

48 healthy adult volunteers (25 Female, median age = 29 years, range = 18–63 years) were recruited to this double-blind, counterbalanced, repeated measures study. We did not perform a formal sample size calculation, as there was no prior data to base a power calculation upon. As our previous study (Finnegan et al. 2023) used discordance in both directions and utilised considerably larger gradients than the present study. Furthermore, as Finnegan et al. (2023) was underpowered to detect individual differences, a considerably larger sample was recruited in the present study. Participants were recruited via University mailing lists or social media advertisements and were invited to two in-person visits at the clinical trials unit at the Oxford Centre for Diabetes Endocrinology and Metabolism (OCDEM), Oxford. Exclusion criteria included significant cardiac, neurological, metabolic, or psychological disease, history of cardiac tachyarrhythmia, prescription and non-prescription drug dependency, history of smoking, and conditions leading to breathlessness such as asthma or COPD. Participants were required to be able and willing to ride a bicycle. Written informed consent was obtained from all participants, and all procedures were conducted in accordance with the Declaration of Helsinki. The study was granted ethical approval by University of Oxford Central University Research Ethics Committee (Ethics Ref: R68447/RE001).

#### Questionnaires

To investigate any psychological and mood related effects on inter-individual differences, participants answered the following questionnaires at the beginning of their first visit: State-Trait Anxiety Questionnaire (Spielberger, 1971), Centre for Epidemiological Studies Depression Scale (CES-D) (Radloff 1991), Anxiety Sensitivity Index (Reiss, 1986), Health Anxiety Inventory (HAI) (Salkovskis et al. 2002), Multidimensional Assessment of Interoceptive Awareness v.2 (MAIA-2) (Mehling et al. 2018), Breathlessness Catastrophising Questionnaire (Solomon et al. 2015), Breathlessness Vigilance and Awareness Scale (McCracken 1997), Positive and Negative Affect Scale (PANAS) (Watson et al. 1988), Fatigue Severity Scale (FSS) (Krupp, 1989a ), and Fatigue Scale (Taylor et al. 2000). Participants then completed the Presence Questionnaire (PQ) (Witmer, 1998) and Simulator Sickness Questionnaire (SSQ) (Kennedy, 1993; Sevinc and Berkman 2020) after cycling to assess immersion and any adverse effects of the paradigm. Individual questionnaire descriptions are in Appendix 1.

#### Virtual reality and cycling protocol

**Fig. 2:**
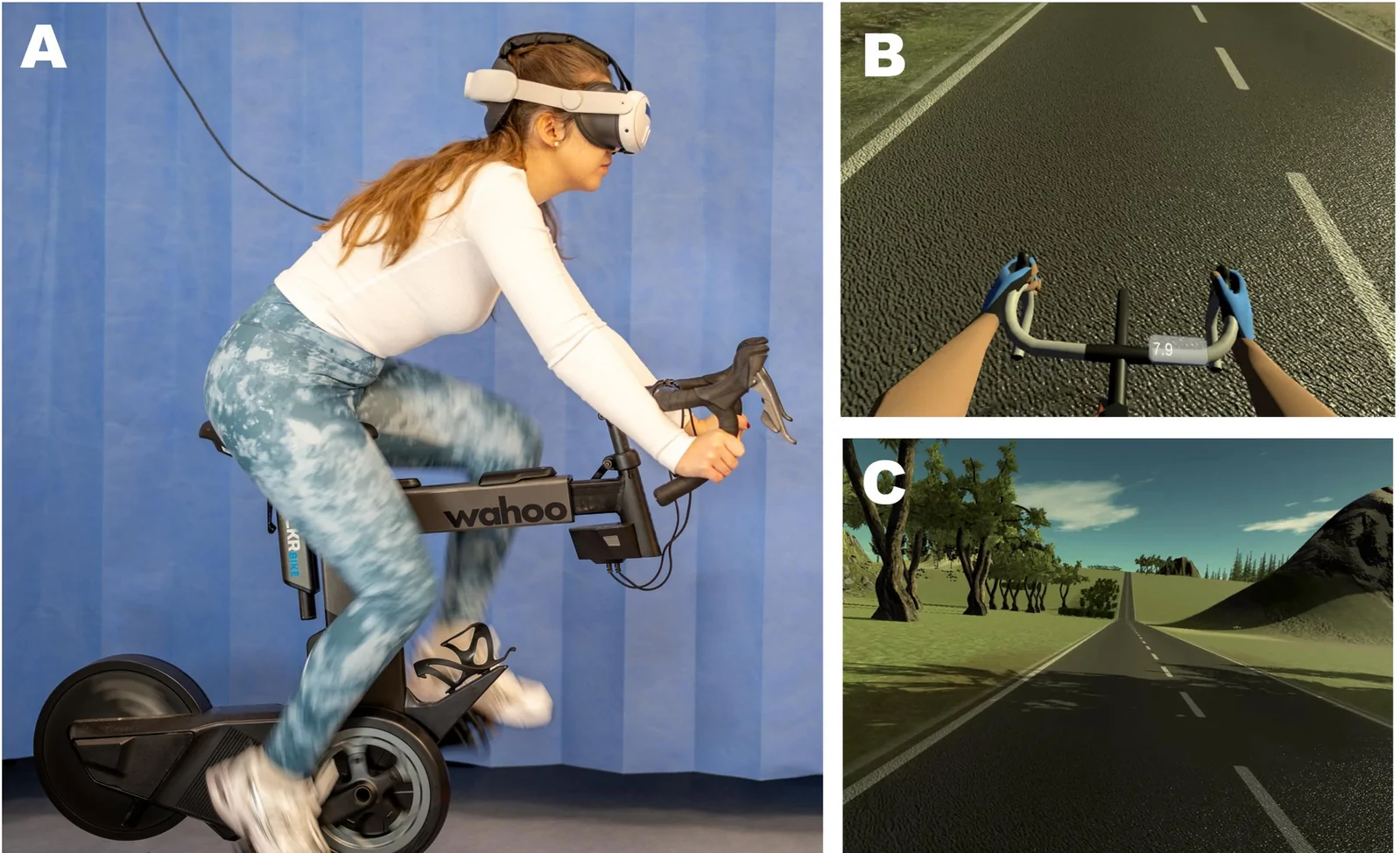
**A**: VR cycling paradigm: participant is riding on the Wahoo KICKR bike at flat 0° angle wearing a Meta Quest 3 VR headset (wire suspended so not to be noticed by the participant during riding). **B**: view of handlebars in VR from rider’s perspective and **C**: view of the virtual environment with hill in distance

The virtual reality cycling programme was created using Unity (version 2019.2.17) and is described in Finnegan et al. (2023) and in Appendix 2. The programme allowed a virtual cycling course to be displayed in real time to an Oculus Rift S headset (Meta Platforms Inc., USA.), or any Steam VR enabled headset. The speed at which the participant moved through the virtual environment was controlled by their real-life pedalling on a stationary bicycle (Wahoo KICKR BIKE (Wahoo Fitness LLC, USA) used with factory calibration) that connected via ANT + to a Razer Blade 15 laptop (Razer Inc., USA/Singapore) running Windows 11 (Microsoft Corporation, USA), shown in Fig. 2. Participants were asked to maintain a consistent, self-selected cadence throughout the course exercise ‘blocks’ (described below), which was visible to both the participant and the experimenter. In order to maintain this cadence, participants were able to change gears on the bike. A Wahoo KICKR HEADWIND fan was positioned in front of the bike and set to speed 1, though this was increased if the participant asked. Participants wore a Wahoo TICKR FIT heart rate armband and heart rate was recorded throughout the duration of the exercise sessions. The VR controllers were used to position the VR camera so that it matched the position of the virtual cyclist’s head and hands, to give the correct perspective.

**Fig. 3:**
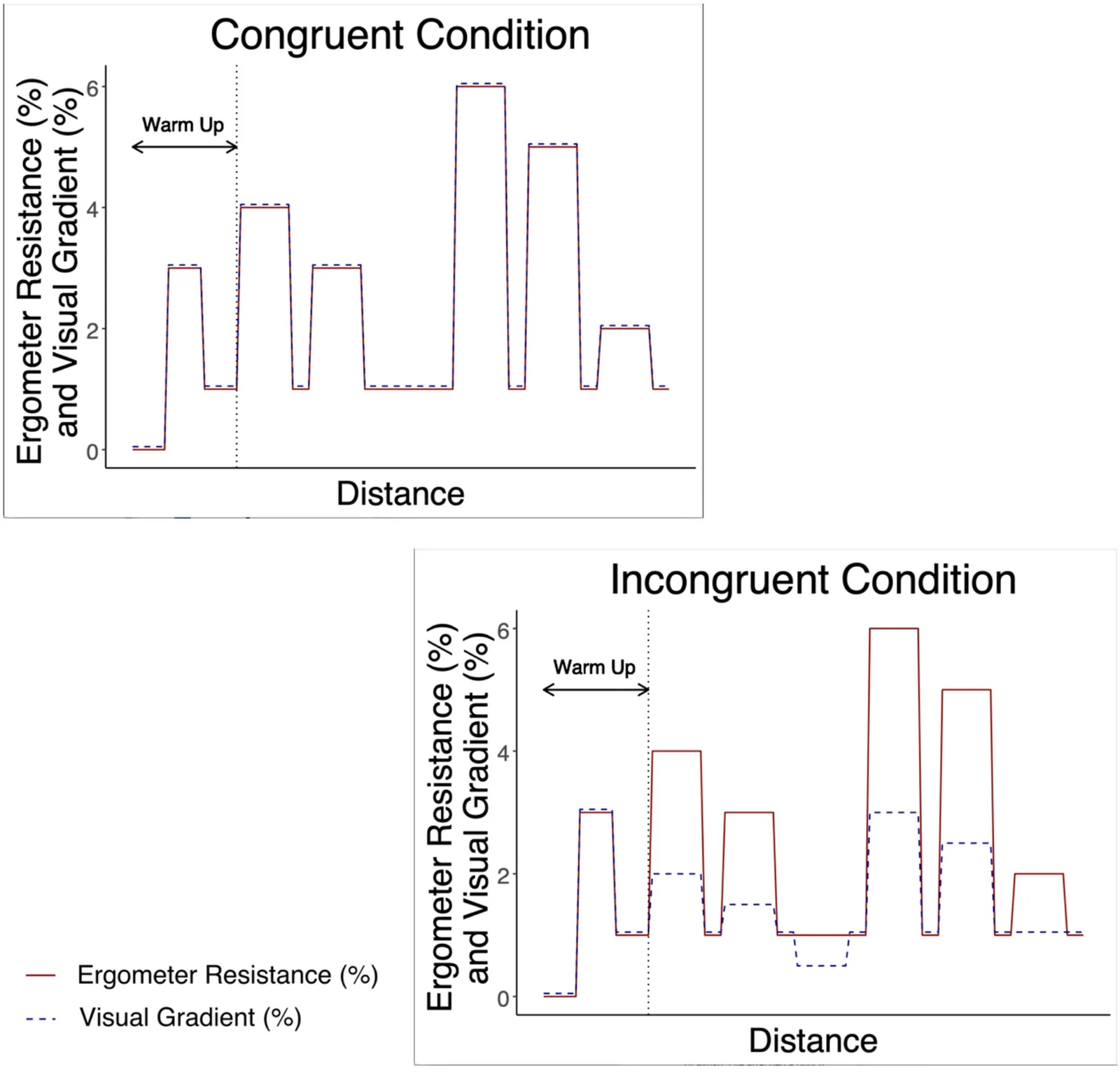
Pedal resistance and visual gradient (solid red and blue dotted line, respectively) between the congruent and incongruent conditions. The order of these hills and pairings were randomly generated for each participant

Participants completed a short ‘warmup’ cycling exercise of three 200 m hills with pedalling resistance and visual slope gradient of 0%, 3% and 1% respectively and in that order. They then completed two exercise blocks of cycling through the virtual terrain. These blocks began with the same three 200 m hills as the warmup, then continued with six 300 m hills in a randomised order with pedalling resistances of 1, 2, 3, 4, 5, and 6%. Visual slope differed depending on condition and was either the same as the physical pedalling resistance (congruent) or approximately 50% lower than the pedalling resistance (incongruent). Figure 3 shows the pairings of pedalling resistance with visual hill slope between the congruent and incongruent conditions. The order of the conditions was hidden from both participants and the experimenter in a double-blind design. One author, independent of data collection and analysis randomised the files to determine slope order. This involved using a random sequence generator to rename the files with random codes. The unblinding list was kept inaccessible to the researchers conducting the data collection. Unblinding happened after the data had undergone pre-processing. Part of the computer screen was covered to ensure that the experimenter was unaware of which courses were congruent and which were incongruent. Participants cycled either the congruent course in their first visit or the incongruent course, with the order determined at random in advance of the study.

At the end of each 300 m hill participants were presented with a 50 m section of 1% (both visual and pedalling resistance). During the final 25 m of the 300 m hill, a visual question appeared on screen, and the experimenter also verbally asked about their level of breathlessness (“How breathless are you from 1 to 10?” where 1 is the lowest) and leg fatigue (“How tired are your legs?” from 1 to 10, where 1 is the lowest). The experimenter provided no other cues during the exercise blocks. After each exercise block the participants dismounted the bike to complete the Presence Questionnaire and the Simulator Sickness Questionnaire and had a 10-minute break before the next exercise block began. The second exercise block proceeded in the same way as the first, with the order of the six 300 m hills randomised, followed by the two questionnaires at the end.

The second visit took place no more than 6 weeks later and replicated the first visit. The State Anxiety Inventory questionnaire was repeated and a custom exercise questionnaire assessed how active participants’ lifestyles were.

#### Data analysis

Data was analysed using R v.4.3.3. Data was condensed by taking the mean of each pedalling resistance (1–6) in each condition (congruent or incongruent) for each participant. The data was tested for normality using Shapiro-Wilk tests, corrected for multiple comparisons using Bonferroni corrections. This showed all variables were non-normally distributed, so non-parametric equivalents of statistical tests were used where possible in the analysis. To carry out these analyses, continuous variables were centred and scaled. When creating linear mixed effect models for analysis, the lmer package in R was used. Variables were selected based on our previous work (Appendix 3). As power, pedalling resistance, and heart rate are highly correlated with each other, only power was included in the model. A LASSO regression was performed to identify questionnaires for inclusion in the linear model (Appendix 4). Given that the SSQ mostly measures physical effects that are also common symptoms of exercise, these results were not used in the regression modelling. However, to assess if there was a difference in VR immersion between congruent and incongruent sessions, a Wilcoxon signed-rank test was performed on SSQ and, separately, PQ scores between these conditions.

### Results

#### Participant demographics

Fourty-eight participants were recruited, with 3 excluded for not following cycling protocol or not completing the course, leaving 45 being used in the final analysis. Table 1.

**Table 1:** Participant demographics and questionnaire scores

*N* = 45	Score	Clinical threshold (where applicable)	*N* Above clinical threshold
Median lin years (± range)	29 (20.5)	–	–
Gender (female)	25	–	–
BMI (kg/m^2^ ± SD)	30 (3.3)	–	–
Days between visits (mean ± SD)	15 (14)	–	–
Interoception (MAIA total) (mean ± SD)	23 (5)	–	–
State anxiety (mean ± SD)	32 (9)	37+ (Kayikcioglu et al. 2017)	13
Trait anxiety (mean ± SD)	36 (9)	37+ (Kayikcioglu et al. 2017)	17
Depression (CESD) (mean ± SD)	16 (5)	17+ (Jiang et al. 2019)	14
Anxiety sensitivity (ASI) (mean ± SD)	15 (10)	–	–
Health anxiety (HAI) (mean ± SD)	12 (7)	18+ (NHS 2024)	8
Breathlessness catastrophising (BC) (mean ± SD)	6 (8)	–	–
Breathlessness awareness (BVAS) (mean ± SD)	27 (12)	–	–
Positive affect (PANAS) (mean ± SD)	34 (6)	–	–
Negative affect (PANAS) (mean ± SD)	17 (5)	–	–
Fatigue severity (FSS) (mean ± SD)	3 (1)	36+ (Taylor et al. 2000)	0
Fatigue scale (mean ± SD)	3 (3)	–	–
Simulator sickness (SSQ) (mean ± SD)	52 (10)	–	–
Presence (PQ) (mean ± SD)	23 5)	–	–

#### Questionnaires

Questionnaires that contributed most to breathlessness and leg fatigue were identified via LASSO regression. When leg fatigue was examined, the questionnaires identified were the CESD depression scale and the Health Anxiety Inventory (HAI). Only the CESD was identified for breathlessness. These selections were then included in subsequent linear models.

#### Physical work increases with pedalling resistance

**Fig. 4:**
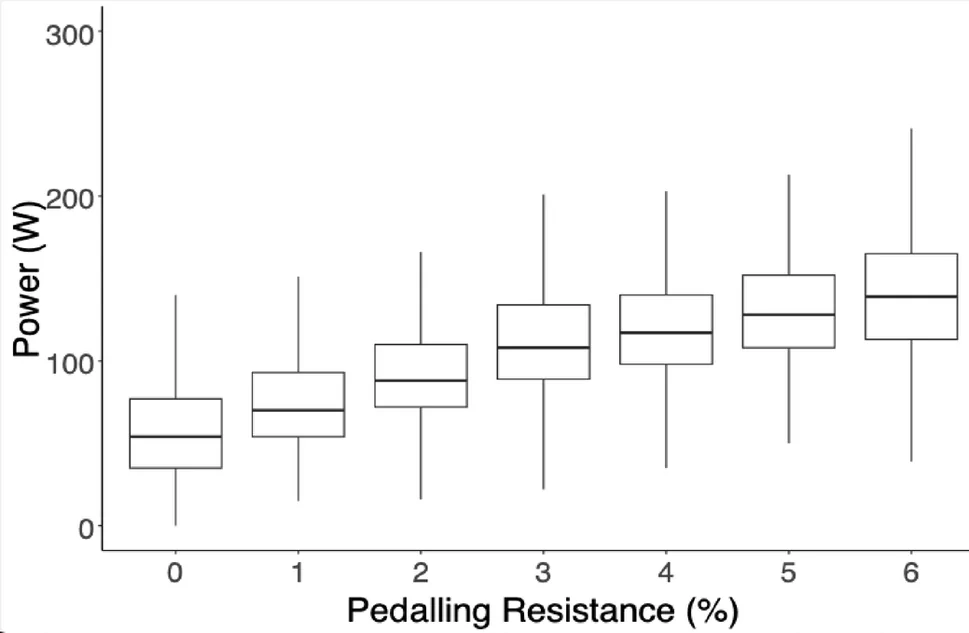
Box plot of power (W) variation related to pedalling resistance (%). Boxes depict the median (horizontal line) within quartiles 1–3 (bounds of box). Whiskers represent 1.5 times the IQR. All pairwise comparisons were significantly different from each other (*p* < 0.001)

As our study concerns the difference in perceived versus actual work effort whilst cycling, we first tested the assumption that cycling against higher pedalling resistance would output more power. A Kruskal-Wallis test was performed to confirm that power (W) changes across levels of pedalling resistance (%) (*p* < 0.001). A clear linear relationship was identified, allowing power to be used to represent ‘actual work effort’ when modelling effort perceptions (Fig. 4).

#### No significant reduction in immersion during incongruent VR

To determine if incongruent VR stimulation increased any sensations of simulator sickness or decreased participants’ presence in the virtual environment, a Wilcoxon signed-rank test was performed on both SSQ and PQ scores between congruent and incongruent cycling sessions. No significant difference in scores from either questionnaire was found between conditions (SSQ: w = 412, *p* = 0.764; PQ: w = 358, *p* = 0.489), suggesting that incongruent VR in our protocol did not affect participants’ immersive experience.

#### Visual slope is an independent predictor of breathlessness and leg fatigue

**Fig. 5:**
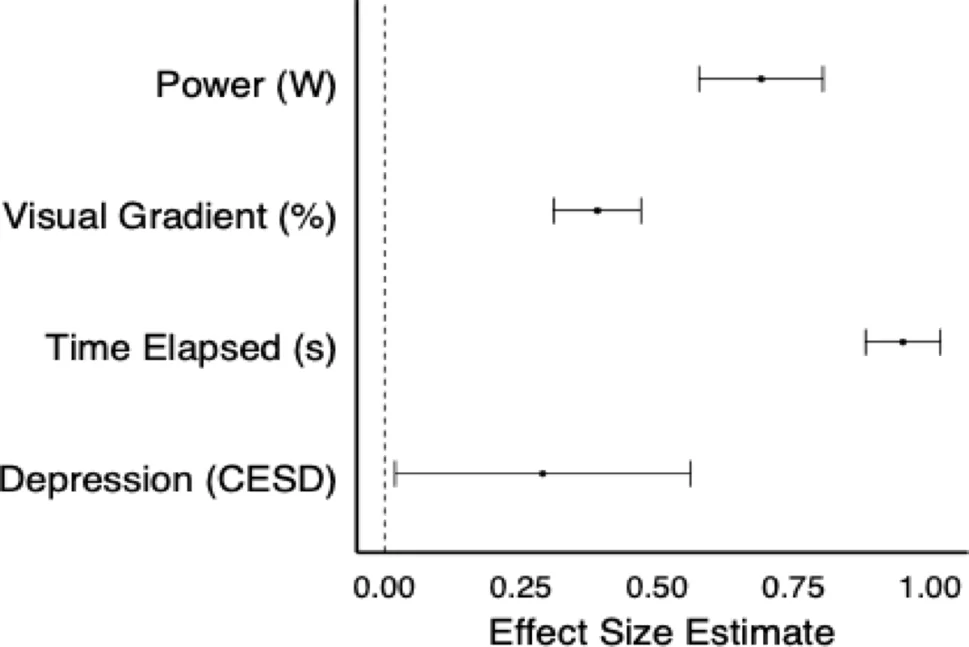
Forest plot showing effect sizes of power, visual gradient, time elapsed and mood as predictor variables for feelings of breathlessness

**Fig. 6:**
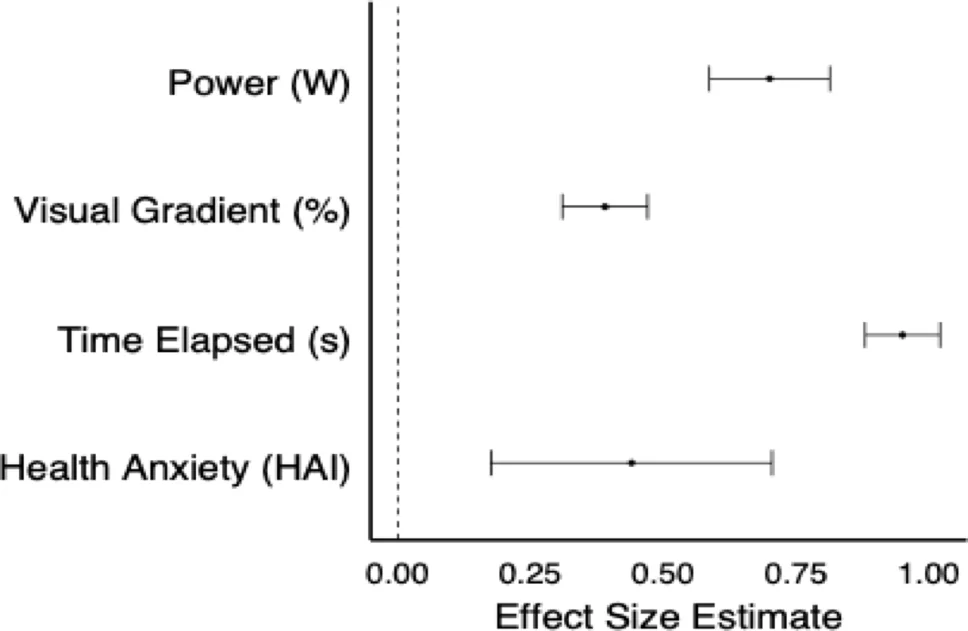
Forest plot showing effect sizes of power, visual gradient, time elapsed and mood as predictor variables for perception of leg fatigue

Two mixed-effects linear models were created, as outlined in the methods, to explain breathlessness and leg fatigue (full models in Appendix 4). Following model optimisation by removing non-significant terms, we found that, accounting for covariates, the visual slope gradient (%) an individual observed was positively associated with both breathlessness and leg fatigue (Tables 2 and 3; Figs. 5 and 6). As predictor variables were scaled before modelling, effect sizes below indicate the change in breathlessness or leg fatigue on a 1–10 Likert scale elicited by one standard deviation change in that predictor variable. This shows that, on average, as visual slope increased, participants perception of their breathlessness and leg fatigue also increased, independent to actual pedalling resistance. Other covariates that were positively associated with these effort perceptions were power (W), which was related to pedalling resistance, and time-elapsed of cycling. Interestingly, scores on the depression-based questionnaire CESD and health anxiety questionnaire HAI were both positively associated with breathlessness and leg fatigue, respectively (also Tables 2 and 3; Figs. 4 and 5).

**Table 2:** Effect size estimates, standard error and p values for the contribution of each predictor variable to breathlessness

Variable	Effect size estimate	Standard error	*P* value
Power (W)	0.69	0.058	< 0.001
Visual gradient	0.39	0.041	< 0.001
Time elapsed	0.95	0.035	< 0.001
Depression (CESD)	0.29	0.138	0.04
Intercept	3.52	0.14	< 0.001

**Table 3:** Effect size estimates, standard error and p values for the contribution of each predictor variable to leg fatigue

Variable	Effect size estimate	Standard error	*P* value
Power (W)	0.70	0.058	< 0.001
Visual gradient	0.39	0.041	< 0.001
Time elapsed	0.95	0.036	< 0.001
Health anxiety (HAI)	0.44	0.135	0.002
Intercept	3.54	0.132	< 0.001

### Discussion

#### Incongruent VR attenuates feelings of breathlessness and leg fatigue

We found a significant attenuation effect on perceived breathlessness and leg fatigue when visual slope was lower than pedalling resistance in comparison to when visual slope was appropriate for pedalling resistance. This supports our previous work and demonstrates that, first, such an effect remains in a larger sample size and, second, it extends to another sensation of exertion: leg fatigue. These results fit into a growing body of research exploring the potential benefits of VR exercise. For example, one recent study showed that participants completing a boxing exercise game in VR exerted more effort than when completing the same exercise game in front of a flat screen (Barbour et al. 2024). Other work has demonstrated, similarly, that people exercising in VR find it more enjoyable and doing so may lead to an increase in exercise habit-forming (Mouatt, 2020). Such results seem particularly pronounced in immersive VR and are likely due to the powerful distraction a virtual-interactive environment may provide from uncomfortable sensations of physical exertion (Li et al. 2011). Whilst the benefits of exercising in VR have so far focused on the role of immersion and distraction, our paradigm advanced this and found that manipulating virtual environmental cues affected participants perception of their real life breathlessness and leg fatigue, without significant changes in immersion. This suggests that the attenuation we found in breathlessness and leg fatigue was a direct result of the incongruent stimuli and not merely the VR environment providing a distraction-mediated analgesia.

#### Psychological factors may mediate effect

In the main study, we found that depression (CESD) and health anxiety (HAI) scores both showed a correlation with perceptions of breathlessness and leg fatigue. This is consistent with previous literature focused on the relationship between mood indicators and interoception. Specifically, research has suggested that breathlessness perception may arise from a combination of incoming nervous signals and individuals’ expectation (Marlow et al. 2019), which is itself informed by internal factors such as prior experience and mood. When seeking to optimise the benefits of VR, previous research has measured participants ‘presence’ in VR, with VR success linked to higher presence (Michael Wilkinson, 2021; Weber et al. 2021). It is therefore promising that our results found no significant decrease in presence during incongruent VR cycling. However, some studies have found that individuals who express certain personality traits, like openness, neuroticism and extraversion, often report higher scores of presence (Ana Sacau, 2008; Weech et al. 2019; Weibel et al. 2010). It follows that an individual’s psychology may influence how susceptible they are to our paradigm. Despite finding that CESD and HAI scores are significant predictors for breathlessness and leg fatigue perception, respectively, this was highly variable, in part due to our healthy cohort. However, psychological factors may play a larger role in susceptibility to our VR paradigm and the physical sensations it could manipulate in clinical populations. For example, people with chronic breathlessness often score higher on depression and anxiety indices, such as the CESD (Blazer and Hybels 2010).

## Patient and Participant Involvement (PPI)

### Introduction

Our work has provided significant evidence that incongruent VR stimulation can attenuate feelings of exertion in healthy participants. To explore the clinical applications of these findings we then completed extensive PPI with relevant stakeholders. This was partly to gauge enthusiasm for such a VR cycling paradigm in a therapeutic context as well as to gain valuable insight into the lived experience of patients with chronic breathlessness and a wider understanding of exercise rehabilitation programs from the perspectives of both users and providers.

### Methods

#### Ethics

According to the NIHR-established national advisory group INVOLVE, formal ethical approval is not required to conduct PPI consultations aimed to explore opinions on research proposals, because the participants are acting as specialist advisors in planning and design of research (NIHR, 2024). However, it was ensured that data protection was enacted according to the General Data Protection Regulation 2018 and that all necessary actions were undertaken to protect confidentiality and anonymization of participants in line with Good Clinical Practice (Goddard 2017).

#### Participants

66 participants total, consisting of patients and healthcare providers, 33 women and 33 men, with an age range of 36–94, shown in Table 4.

**Table 4:** Participant demographics

Patients		
Number	Recruited from	Sex (M: F)
19	British lung foundation breathe easy group (SE England)	5:14
10	Cardiac rehabilitation (James cook hospital, middlesbrough)	10 M
7	Cardiac clinic (Hull York Medical School)	5:2
5	NIHR diversity in research PPI group (Oxford Biomedical Research Centre)	2:3
4	Cardiac rehabilitation (Oxfordshire services)	3:1
Healthcare professionals		
Number	Occupation	Sex (M: F)
10	Cardiac physiotherapist (Oxfordshire, reading, middlesbrough)	2:8
2	Pulmonary physiotherapist (Oxfordshire)	1:1
2	Pulmonary rehabilitation assistant (Oxfordshire)	2 F
2	Cardiac nurse (Middlesborough)	2 F
3	Clinical leads: complex rehabilitation (York), cardiac and pulmonary rehabilitation (Oxfordshire)	3 M
2	Cardiologists (York, middlesborough)	2 M

#### Session structures

Sessions were a mix of focus groups and one-to-one interviews. In all of them, participants were given context for the project background, followed by a demonstration of the bike, in which they all cycled for a few minutes with the VR headset, before engaging in a longer discussion about their views on the research idea of using virtual reality to help enhance exercise-based rehabilitation. Sessions lasted between 60 and 120 min.

### Results and discussion

#### Incorporating immersive VR into exercised based rehabilitation

Cardiac and pulmonary rehabilitation programmes are successful therapies for a range of cardiorespiratory conditions (Rochester et al. 2015; Taylor et al. 2022). In the UK, these are short term supervised exercise classes, often lasting 10–12 weeks and taking place in hospital or commercial gymnasiums. Of the patients we spoke to who had completed cardiac rehabilitation, six out of fifteen described the gym environment as ‘intimidating’. Indeed, cardiac physiotherapists who facilitated rehabilitation within their hospital suggested this setting may indicate to patients that exercise is much riskier than it is, potentially limiting exercise take-up outside of sessions. Patients were interested in testing whether a VR cycling paradigm could help alleviate these anxieties, by providing an ‘escape’ from the actual rehabilitation location. Physiotherapists also noted a marked recovery improvement when participants were able to complete additional exercise, which echoes the literature (Dibben et al. 2023). However, patients that didn’t live near green spaces, or felt unsafe walking outside their home, were much less likely to complete the recommended weekly exercise. This provides another opportunity for VR to help, in creating a home-based therapy that immerses users in virtual green spaces. Finally, after completing their rehabilitation programme, participants felt that barriers to exercise can significantly increase, and this may be a further application of such technologies. Particularly, whether such a portable VR cycling tool may help with exercise uptake after a supervised programme ends and gym attendance becomes more difficult.

#### Considerations for the application of a VR cycling paradigm in cardiorespiratory rehabilitation

Whilst there is indeed a clear therapeutic potential, target for application and excitement around such a VR cycling paradigm, adjustments would need to be made to the protocol used in this paper. In particular, although all 41 patients were able to cycle, there were considerable accessibility barriers in our current set up. For example, the Wahoo KICKR bike requires a forward, low riding position, which was uncomfortable for those unfamiliar with cycling. Whilst most cardiac patients who had been through rehabilitation could tolerate the forward posture, any pose that compacts the chest cavity may be unsustainable for patients with breathing difficulties. Further, the presence of a crossbar ensured many participants required a step stool and physical assistance to mount and dismount the bike frame, thus requiring users to possess greater flexibility and balance then they would need to simply sit and pedal. Importantly, an identified benefit of in-person rehabilitation sessions was the opportunity to socialise. Several physiotherapists were concerned that a VR headset may act as a physical barrier to social engagement in-person, but wondered if a future study might clarify this, for example, if patients fared better when other cyclists were present in the virtual environment.

## Study two: feasibility study of tolerance and acceptability in people with cardiac and respiratory conditions

### Introduction

After finding widespread enthusiasm for such a VR cycling paradigm in patient groups and with exercise rehabilitation facilitators, we then conducted a small tolerance and acceptability study in a small cohort of participants with cardiac and respiratory conditions. As the purpose of this was to pilot feasibility of such a VR cycling paradigm in a clinical context, the study protocol was designed to mirror similar exercise regimes in cardiac rehabilitation. Figures 7, 8.

### Methods

**Fig. 7:**
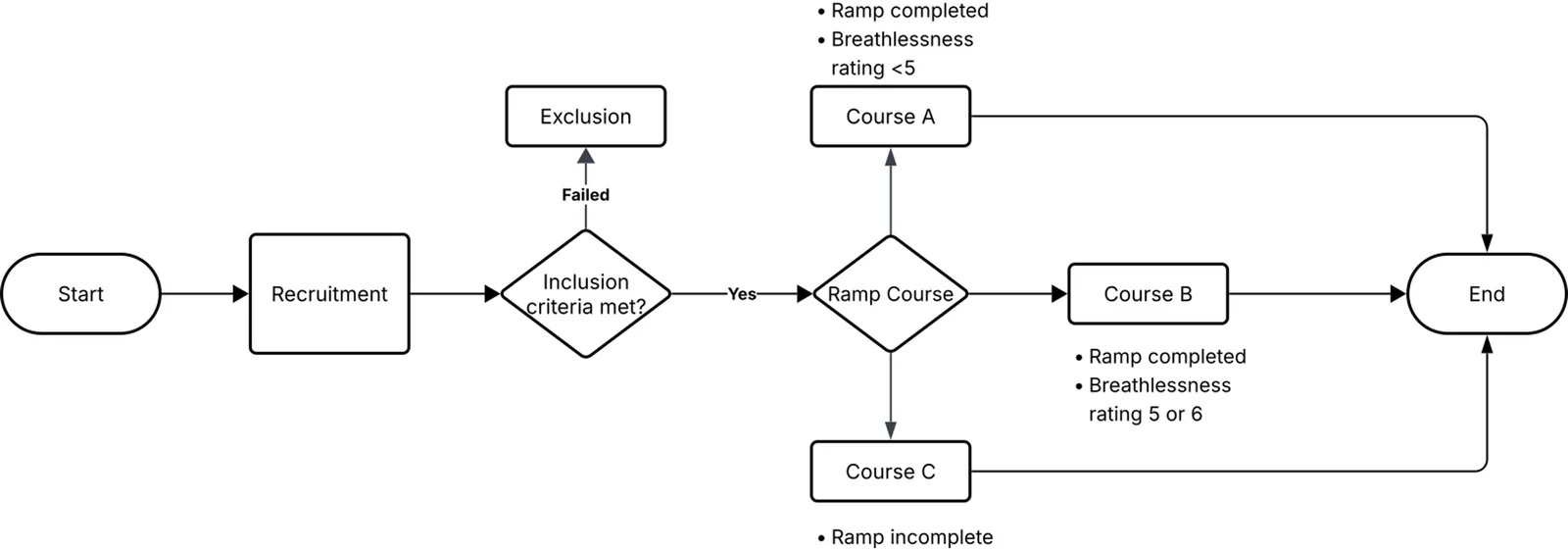
Flow chart of study two, from recruitment to data collection, showing course difficulty assignment

#### Participants

Nine patients participated in the study, with seven being able to cycle the test course to completion. Participants had an age range of 61–76 and mean BMI of 28.7, demographics and questionnaire scores in Table 5. Prior to the study, participants underwent a phone screening to ensure eligibility. Exclusion criteria included: unstable angina, aortic stenosis, recent embolism, uncontrolled diabetes, resting blood pressure above 180/100, uncontrolled atrial/ventricular arrythmias, full list in appendix (appendix 5). These criteria were set based on guidelines published by the Association of Chartered Physiotherapists in Cardiovascular Rehabilitation (ACPCR) (Inbar et al. 1994).

**Table 5:** Participant demographics and questionnaire scores

N = 9	Score	Clinical threshold (when applicable)	N above clinical threshold
Median age in years (± range)	68.5 (15)	–	–
BMI (kg.m^− 2^ ± SD)	28.7 (2.61)	–	–
General anxiety (GAD-7) score (mean ± SD)	8.2 (1.70)	5–9 = mild anxiety (Spitzer et al. 2006)	9
Dyspnoea (DYS-12) Score (mean ± SD)	12.90 (2.66)	–	–
Depression (PHQ-9) score (mean ± SD)	9.81 (1.19)	5–9 = mild depression (Kroenke et al. 2001) 10 + = moderate	5 4
Fatigue (FSS-10) score (mean ± SD)	26.75 (5.58)	36+ (Taylor et al. 2000)	1
Breathlessness catastrophising (BC-13) Score (mean ± SD)	1.33 (2.47)	–	–
Gender-Female	1	–	–

#### Questionnaires

To observe the interaction between baseline mood and perceived breathlessness, participants completed a short battery of questionnaires in line with previous studies (Finnegan et al. 2023). These were completed before exercising. Mental wellbeing was assessed using General Anxiety Disorder-7 (GAD-7) (Spitzer et al. 2006), Patient Health Questionnaire-9 (PHQ-9) (Kroenke et al. 2001) and Fatigue Severity Scale-10 (FSS10) (Krupp et al. 1989a ) and breathlessness was assessed using Dyspnoea-12 (DYS12) (Yorke et al. 2011) and Breathlessness Catastrophising-13 (BC13) (Solomon et al. 2015).

#### Health assessment

A medical assessment to assess suitability for exercise was undertaken by a suitably trained clinician following ACPCR guidelines. A maximum heart rate threshold was calculated according to ACPCR guidelines, above which participants would be asked to terminate the test.

#### Experimental set up

The same bike, laptop and software were used as outlined in Sect. 2.2.3. This study, however, used the more up-to-date Meta Quest 3 VR headset. Bike seat height, handlebar height and crossbar length were all adjusted to be comfortable for each participant.

#### Ramp course

As this cohort was of more variable health and prone to breathlessness, they first completed a ramp course, the results of which would determine the difficulty of test course they received. This is designed to echo the protocol used at the start of cardiac rehabilitation, where results of a ramp test completed on cycle ergometers are used to personalise an exercise program according to capacity. In our study, participants would cycle in VR, as outlined previously, along virtual hills whereby pedalling resistance and visual slope increased, concordantly, every 450 m. Throughout the study, the Wahoo KICKR was in ‘bike’ mode rather than ‘ergometer’ mode and as such, estimations of power (W) relative to resistance were taken from the power output of cycling against those pedalling resistances at a stable cadence of 60 rpm. The pedalling resistance increases in the ramp course were then chosen to approximately match those used in cardiac rehabilitation ramp tests, whereby power output increases, stepwise, in 15-watt increments (Table 6). At the end of each resistance level, participants were asked to rate their breathlessness and, separately, leg fatigue on a 1–10 Likert scale, as in study one. The course was stopped either when participants completed the course, achieved a breathlessness of six or reached their heart rate threshold (outlined above, full equations in Appendix 6), whichever occurred first. If participants could complete the course with a breathlessness rating of < 5, they would cycle test course (A) If they completed the course but reached a breathlessness rating of 5 or 6, they would cycle test course (B) Finally, if the ramp was stopped before completion, they would cycle test course C.

**Table 6:** Ramp course

Distance (M)	Pedalling resistance (% gradient)	Visual slope (% gradient)	Approximate power (W) at a cadence of 60
50	0	0	30
450	1	1	45
450	1.5	1.5	60
450	2	2	75
450	2.5	2.5	90
450	3	3	105

#### Test course

Participants then cycled a course in the same virtual environment consisting of twelve 150 m slopes, where visual gradients were generated independent to pedalling resistance. To make the study more accessible, we asked participants to only cycle one test course. Therefore, we adapted courses from our previous work rather than from the two-session design of study one in this paper (Finnegan et al. 2023). For each virtual hill, visual slope was either congruent to, lower than or higher than pedalling resistance and the order of these slope-resistance pairings was randomly generated per participant. We then stratified these courses into three difficulty levels that patients would be assigned based on the results of the ramp course described above. Test course A had a maximum visual slope of 4% and a maximum pedalling resistance of 3%, test course B was 50% of A and test course C was 25% of A (Fig. 6, example courses in Appendix 7). Per previous studies, patients rated their breathlessness and leg fatigue, separately, on a 1–10 Likert scale after each slope. Participants were asked to keep a comfortable cadence as consistently as possible.

**Fig. 8:**
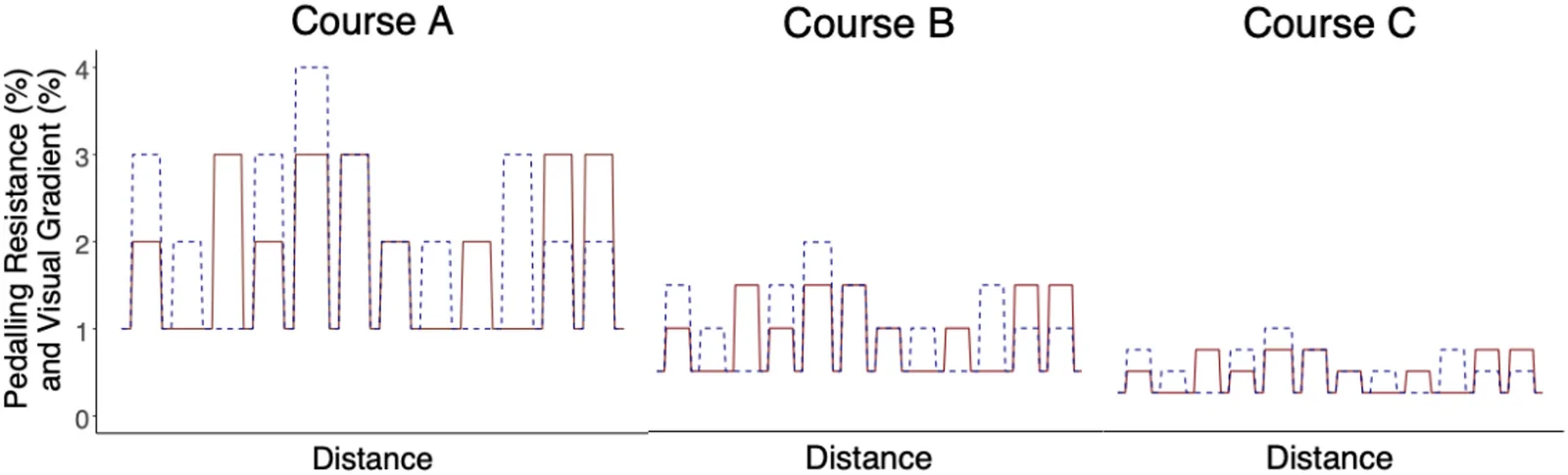
Shows pedal resistance and visual gradient (dotted blue and solid red lines, respectively) pairings in the patient study, stratified across course types. The order of these slopes were randomised for each participant

#### Data analysis

Data was analysed using R v.4.3.3 and following the same protocol as study one. As above, a linear mixed effect model was created to predict breathlessness and, separately, leg fatigue ratings. However, given our restricted sample size, these models should be considered exploratory and as such are found in the appendix (Appendix 8).

### Results and discussion

#### Tolerance and acceptability of VR cycling paradigm in patients

The mean cycling time during the test course was 10 min and 21 s (SD = 2 min and 53 s). Five participants cycled the highest intensity level of course (A), two cycled the medium level (B) and two cycled the lowest intensity level (C). The participants that cycled course level C did not complete. This demonstrates that the variability in physical ability and reinforces the requirement to include personalisation in course difficulty for future therapeutic tools. Despite differing needs of individual participants, our feasibility study showed that many were able to tolerate the paradigm once suitably adjusted for each individual. The end goal of both cardiac and pulmonary rehabilitation courses is for patients to be able to do 30 continuous minutes of aerobic exercise, such as walking, daily (Mytinger et al. 2020). Both the ramp and test course, when completed, lasted for approximately 15 min each, which most patients were able to cycle for without reaching six out of 10 on a breathlessness scale. This suggests our difficulty levels are reasonable. None of this cohort experienced any adverse effects of the VR and were unable to notice a difference between congruent and incongruent slopes. Finally, though none of these participants had used a VR headset before, this new technology was immediately accepted and most found the cycling experience enjoyable and more interesting than a standard exercise bike.

## General discussion

### Overview

The purpose of this paper was to further explore the effects of incongruent stimulation within our VR cycling paradigm and investigate the therapeutic potential of such a tool in relevant patient groups. After finding a similar attenuation effect of breathlessness, and now leg fatigue, in a larger sample of healthy participants, we interviewed a group of relevant patients and healthcare professionals. Finally, we completed a small tolerance and acceptability study in people with cardiac and respiratory conditions. This work follows the recommendations of an international working group on how to approach VR research in healthcare (Birckhead et al. 2019). Their study outlines three stages for clinical research into VR: VR1—content development by working with patients, VR2 – early testing with a focus on tolerability and acceptability and VR3—randomised controlled clinical trial.

### Therapeutic potential of VR in cardiac and pulmonary rehabilitation

#### Barriers to rehabilitation

The British Heart Foundation recommends people with cardiopulmonary conditions do 150 min of moderate aerobic exercise a week (BHF 2024). However, with many people unable to meet this threshold, novel ways to reduce barriers to exercise are urgently needed and VR may be a valuable tool to help. A 2022 scoping review identified both the environment (such as season and weather) and lack of motivation as two key barriers to exercise in people with COPD (Xiang et al. 2022). Studies on exercise within cardiovascular patients find similar barriers, along with reporting a fear of overexertion, especially in patients with heart failure (Çakal et al. 2022; Farris et al. 2019). In regard to fear, studies have reported that reassurance from a clinician along with delivery of specific information about the safety of exercise is beneficial, highlighting the importance of supervised exercise (Okwose et al. 2020). However, physiotherapists interviewed as part of this work also suggested some people are intimidated by the hospital environment and as a result are less likely to complete rehabilitation. These barriers contribute to the considerable dropout rate for rehabilitation programs. One clinical lead for pulmonary rehabilitation stated that they estimate 25% of the cohort drop at each stage of the pathway, an equivalent of 57% dropout from referral to final session. This is consistent with a recent narrative review that found dropout in higher-income countries to be as high as 56% (Turk-Adawi and Grace 2015). A 2014 study in Spain found fewer than 7% of eligible patients participate in cardiac rehabilitation (Resurrección et al. 2018). Within cardiac rehabilitation, patients with heart failure have reduced capacity for physical exercise and take part in separate, non-gym sessions (Bozkurt et al. 2021). These data highlight the strong clinical need for new rehabilitation tools (Kelly Evenson, 2021; Moore et al. 2006). Indeed, many participants preferred using the VR cycling paradigm to equivalent exercise regimens in cardiac and pulmonary rehabilitation programmes. This suggests such a tool may increase engagement with exercise in relevant patients.

#### Potential of exergames

While ‘exergames’ exist, combining exercise and video gaming, none use incongruence in the same way as the present study. In fact, systematic reviews into the use of virtual reality and video games in cardiac rehabilitation have found that most used systems for ‘virtual reality’ were not head mounted displays as in the present study, but instead video game consoles such as the Nintendo Wii (García-Bravo et al., 2021). In these studies, participants enjoyed playing the exergames, which resulted in decreased depressive symptoms and an energy expenditure comparable with light to moderate intensity activity (Klompstra et al. 2014).

Interventions using head mounted virtual reality displays, such as FitXR, a boxing exergame. When compared with playing the same game on a flat, 2D screen, head mounted VR used more oxygen per minute with the same perceived level of exertion (Barbour et al. 2024). This suggests that even without our incongruent manipulation, VR exergames can encourage individuals to work harder without increasing their perceived exertion. An avenue for future research could be to compare such existing exergames with our current intervention on factors such as usability and enjoyment, as well as breathlessness.

#### A personalised therapy

Interestingly, we also find a role for anxiety and depression in modulating susceptibility to our paradigm. If an individual’s psychology does indeed mediate any beneficial effects, psychological profiling may identify users who would most benefit from the VR paradigm. Such profiling could be used in clinic, prior to beginning exercise rehabilitation, to stratify therapeutic pathways for patients. This could both help maximise clinical benefit and increase the personalisation of exercise programs.

### Limitations

This study provides further promising results for the efficacy of such a VR cycling paradigm in healthy participants and demonstrates enthusiasm in relevant patient groups. However, here we address some limitations of this work that should be considered for future study. First, as this study was particularly interested in how perceptions of breathlessness changed, our measures for breathlessness were purely subjective. It would be of clinical interest to know whether such a paradigm could alter objective physiological markers too. Further research could include measurements of participants’ VO2 max, blood lactate levels, ventilation and lung diffusion capacity, for example. Second, it is important to once again note the very limited sample size of our patient study. This work was done to retrieve preliminary quantitative and qualitative data to supplement our PPI using a full cycling protocol to help inform a future study. Third, as outlined previously, the Wahoo KICKR bike requires users to sit in a low riding position (Fig. 1). This positioning, along with the high frame limits the accessibility of such a set up for many patients. Future work should be done on patient-friendly kit, with a particular focus on compatibility with machines already used for cardiac and pulmonary rehabilitation. Finally, there is the interesting question of habituation. It is not known whether this VR cycling paradigm would offer clinical benefit over the span of 6–12 weeks (the length of rehabilitation programs), nor whether the novelty of the ‘trick’ would wear off, leading to habituation with the protocol. Such habituation may alter the effectiveness or level of immersion of the tool. Future work would, therefore, benefit from testing repeated use over a period of time.

Overall, the work of this paper shows there is merit to investigating the potential of an incongruent VR cycling paradigm therapeutically.

## Conclusion

By using a fully immersive incongruent VR cycling paradigm, we have shown that perceived breathlessness and leg fatigue can be driven independently from actual physical effort. These exciting new findings, along with the limitations outlined above, provide ample justification for further research. An important next step to assess clinical benefit and feasibility would be a VR3 level clinical trial. This would allow the formal measurement of sustained changes in exercise tolerance and adherence across the equivalent timescale of a rehabilitation course. Such a trial may also, importantly, elucidate the impact of any habituation effects.

To conclude, not only does this work provide intriguing insight into the powers of VR to manipulate effort perception during exercise but hopefully illuminates a development pathway forward, towards a novel and highly beneficial clinical tool.

## Data Availability

Anonymised data set necessary replicate study findings available here https://osf.io/pa4wt.

## Appendices

**Study One**

***Appendix 1—Questionnaire descriptions***

The State-Trait Anxiety Inventory (STAI) (Spielberger et al. 1971).

40-question scale which is frequently used to measure present moment (‘state’) and longer term (‘trait’) anxiety levels via a 4-point Likert scale (“not at all (1)”, “somewhat (2)”, “moderately so (3)”, “very much so (4)”).

The Centre for Epidemiological Studies Depression Scale CESD (Radloff, 1977).

20 questions regarding depressive symptoms to which participants respond the duration over the past week that they experienced those symptoms: “rarely or none of the time (0)”, “some or little of the time (1)”, “occasionally or a moderate amount of time (2)”, and “most or all of the time (3)”.

Anxiety Sensitivity Index ASI (Reiss et al. 1986).

16 item questionnaire that measures anxiety sensitivity on a 5 point Likert scale as follows: “very little (0)”, “a little (1)”, “some (2)”, “much (3)”, and “very much (4)”.

Health Anxiety Inventory (HAI) (Salkovskis et al. 2002).

Self-report tool designed to measure attitudes towards an individual’s own health. It was designed for use in patients with hypochondriasis and is considered a reliable and valid measure of health anxiety.

Multidimensional Assessment of Interoceptive Awareness (Version 2) (MAIA-2) (Mehling et al. 2018).

Questionnaire with 8 subscales, each capturing a different aspect of interoception – awareness of internal body signals.

Breathlessness Catastrophising Scale (BC) (Bishop and Pivik, 1995; Solomon et al. 2015). used in this study is a 13 item version of the Pain Catastrophising Scale developed but with the word “pain” substituted for the word “breathlessness” Each item is rated on a five-point Likert scale, ranging from “not at all (0)” to “all the time (4)”.

Breathless Vigilance and Awareness Questionnaire (BVAQ), adapted from the Pain Vigilance and Awareness Questionnaire developed by McCracken (1997), substituting the word “pain” for “breathlessness” where appropriate. The questionnaire has 16 items, scored from “never (0)” to “always (5)”.

Positive and Negative Affect Scale (PANAS) (Watson et al. 1988).

Two 10-item subscales, one which captures positive mood over the past week, and one for negative mood. They are scored from “very slightly or not at all (1)” to “extremely (5)”.

Fatigue Severity Scale (FSS) (Krupp et al. 1989).

Self report how much they agree with 9 fatigue-related statements, from “strongly disagree (1)” to “strongly agree (7)”.

Finally, participants were asked to “select a number which describes your global fatigue with 0 being worst and 10 being normal”.

Questionnaires were scored in accordance with their respective manuals.

## Appendix 2—Virtual environment details

The virtual environment was created by using a Unity package containing a single prefab which was entered as a terrain object in Unity. Upon beginning an exercise session, the terrain is set to match the slop values in the configuration file used for that session. Gradients were created visually by changing the height of each terrain height map unit to create a slope.

Using the ANT+ protocol, the Wahoo KICKR bike was connected to the program, which allowed us to set its resistance and retrieve the power output from the user. The resistance was either the same as or different to the visually presented slope (see Fig. 2).

A raycast calculation was performed to measure the observed slope on the terrain from the cyclist’s perspective and was also used in the gravity component to assist in determining the speed of the virtual bike.

## Appendix 3—Linear regression model

Variables for inclusion in the modelling were:

1. informed by those found significant in our previous paper: power, visual slope and BMI.
2. time spent cycling to account for our new design of randomised slope order.
3. and any questionnaires isolated from the LASSO regression.

Model 1 for **breathlessness**: full mixed effects regression model.

Breathlessness ~ Power (W) + Visual Slope (%) + BMI + Time Cycling + CESD + (random participant effect).

**Table:**

*R*^2^_conditional_ = 0.63, *R*^2^_marginal_ = 0.45				
*Fixed effects*	Effect estimate	Std. error	t-value	*p*-value
Intercept	4.7352	0.9697	4.883	< 0.001
Power	0.70069	0.05958	11.760	< 0.001
Visual slope gradient	0.40761	0.04259	9.570	< 0.001
BMI	−0.05170	0.04112	−1.257	0.215
Time spent cycling	0.94923	0.0377	25.180	< 0.001
CESD	0.3649	0.14797	2.466	0.0179

Model 2 for **breathlessness**: optimised mixed effects regression model.

Breathlessness ~ Power (W) + Visual Slope (%) + Time Cycling + CESD + (random participant effect).

**Table:**

*R*^2^_conditional_ = 0.63, *R*^2^_marginal_ = 0.45				
*Fixed effects*	Effect estimate	Std. error	t-value	*p*-value
Intercept	3.528	0.138	25.572	< 0.001
Power	0.6861	0.05776	11.879	< 0.001
Visual slope gradient	0.3991	0.04094	9.749	< 0.001
Time spent cycling	0.9510	0.03551	26.784	< 0.001
CESD	0.2866	0.1384	2.070	0.0442

Model 1 for **leg fatigue**: full mixed effects regression model.

Leg Fatigue ~ Power (W) + Visual Slope (%) + BMI + Time Cycling + CESD + (random participant effect).

**Table:**

*R*^2^_conditional_ = 0.63, *R*^2^_marginal_ = 0.44				
*Fixed effects*	Effect estimate	Std. error	t-value	*p*-value
Intercept	3.648	0.9646	3.782	< 0.001
Power	0.5462	0.05816	9.391	< 0.001
Visual slope gradient	0.3519	0.04133	8.513	< 0.001
BMI	−0.00637	0.04092	−0.156	0.877
Time spent cycling	0.9417	0.03656	25.758	< 0.001
HAI	0.4944	0.1682	2.940	0.0052
CESD	0.1886	0.1681	1.122	0.268

Model 2 for **leg fatigue**: optimised mixed effects regression model.

Leg Fatigue ~ Power (W) + Visual Slope (%) + Time Cycling + HAI + (random participant effect).

**Table:**

*R*^2^_conditional_ = 0.63, *R*^2^_marginal_ = 0.46				
*Fixed effects*	Effect estimate	Std. error	t-value	*p*-value
Intercept	3.538	0.1328	26.637	< 0.001
Power	0.7005	0.05785	12.110	< 0.001
Visual slope gradient	0.3947	0.04092	9.645	< 0.001
Time spent cycling	0.9479	0.03550	26.705	< 0.001
HAI	0.4383	0.1349	3.248	0.0021

## Appendix 4—LASSO regression for questionnaire inclusion in linear model

**Table:**

	s0	
	Breathlessness	Leg fatigue
Intercept	2.713	1.640
State anxiety	.	.
Trait anxiety	.	.
Depression (CESD)	0.017	0.026
Anxiety sensitivity (ASI)	.	.
Health anxiety (HAI)	.	0.005
Breathlessness catastrophising (BC)	.	.
Breathlessness awareness (BVAS)	.	.
Positive affect (PANAS)	.	.
Negative affect (PANAS)	.	.
Fatigue severity (FSS)	.	.
Fatigue scale	.	.
MAIA (Scale 1)	.	.
MAIA (Scale 2)	.	.
MAIA (Scale 3)	.	.
MAIA (Scale 4)	.	.
MAIA (Scale 5)	.	.
MAIA (Scale 6)	.	.
MAIA (Scale 7)	.	.
MAIA (Scale 8)	.	.
Simulator sickness (SSQ) (Total)	0.015	0.031
Presence (PQ) (Total)	.	.

**Study two**

## Appendix 5—Exclusion criteria for patient study

**Table:**

Contraindication
Unstable angina
Resting BP > 180/100 (one or both)
Resting HR > 100 bpm
Aortic stenosis (unless cleared by rehab)
Febrile illness
Uncontrolled atrial/ventricular arrhythmias
Not taking medications as prescribed
Pericarditis/Myocarditis
Uncompensated heart failure
Other untreated metabolic conditions (e.g. Thyroiditis, hypokalaemia etc.)
Recent embolism
Uncontrolled diabetes
Acute thrombophlebitis

## Appendix 6—Health screening protocol

This involved completing a contraindication checklist identical to that used during the phone screening. Participants were then asked some brief questions about their medical history along with any current prescriptions and had their weight and height measured, to calculate Body Mass Index (BMI). Blood pressure and heart rate were taken, and all participants’ blood pressure fell below the exclusion limit of 180/100 along with all resting heart rates below the exclusion limit of 100 beats per minute. The ACPCR ((Inbar et al. 1994) formula was used to calculate the heart rate at which the test would stop for each individual:

1. ***MaxHeartRate = 206 - (0.7 x Age)***.

***If taking beta blockers***,*** −30.***

(2) ***HeartRateReserve = MaxHeartRate – RestingHeartRate***.
(3) ***STOP test at = RestingHeartRate + (0.7 x HeartRateReserve)***.

## Appendix 7—Example test courses for study two

*Twelve test slopes in light grey*,* warm up and inter hill breaks in white.*

**Table:**

Distance (m)	Visual slope			Pedalling resistance		
	A	B	C	A	B	C
150 (warm up)	0	0	0	0	0	0
150 (warm up)	2	2	2	2	2	2
150	2	1	0.5	1	0.5	0.25
50	1	1	1	1	1	1
150	2	1	0.5	3	1.5	0.75
50	1	1	1	1	1	1
150	1	0.5	0.25	3	1.5	0.75
50	1	1	1	1	1	1
150	3	1.5	0.75	2	1	0.5
50	1	1	1	1	1	1
150	2	1	0.5	2	1	0.5
50	1	1	1	1	1	1
150	2	1	0.5	3	1.5	0.75
50	1	1	1	1	1	1
150	2	1	0.5	1	0.5	0.25
50	1	1	1	1	1	1
150	1	0.5	0.25	2	1	0.5
50	1	1	1	1	1	1
150	3	1.5	0.75	3	1.5	0.75
50	1	1	1	1	1	1
150	3	1.5	0.75	1	0.5	0.25
50	1	1	1	1	1	1
150	3	1.5	0.75	2	1	0.5
50	1	1	1	1	1	1
150	4	2	1	3	1.5	0.75
50	1	1	1	1	1	1

## Appendix 8—Linear regression modelling

Our sample size is too low for such modelling to be formally interpreted or included in the main body.

***Effect size estimates***,*** standard error and p values for the contribution of each predictor variable to*** ***breathlessness***.

**Table:**

Variable	Effect size estimate	Standard error	*P* value
Intercept	9.067	2.18	0.0063
Power (W)	0.246	0.105	0.023
Visual gradient	−0.166	0.087	0.062
Time elapsed	0.583	0.069	< 0.001
BMI	−0.207	0.075	0.034

***Effect size estimates***,*** standard error and p values for the contribution of each predictor variable to*** ***leg fatigue***.

**Table:**

Variable	Effect size estimate	Standard error	*P* value
Intercept	6.01	3.26	0.108
Power	0.370	0.128	0.005
Visual gradient	−0.053	0.102	0.609
Time elapsed	0.801	0.080	< 0.001
BMI	−0.097	0.112	0.418

## References

[CR1] Almadana Pacheco V, Pavón Masa M, Gómez-Bastero Fernández AP, Muñiz Rodríguez AM, Tallón Moreno R, Montemayor Rubio T (2017) Patient profile of drop-outs from a pulmonary rehabilitation program. Arch Bronconeumol 53(5):257–262. 10.1016/j.arbres.2016.06.010 27480263

[CR2] Ana Sacau JL, Hartmann T (2008) Influence of individual factors on presence. Computers Hum Behav 24(5):2255–2273

[CR3] Barbour B, Sefton L, Bruce RM, Valmaggia L, Runswick OR (2024) Acute psychological and physiological benefits of exercising with virtual reality. PLoS ONE 19(12):e0314331. 10.1371/journal.pone.0314331 39693283 PMC11654962

[CR4] BHF (2024) Heart Matters: 3 exercises that are best for heart health. Br Heart Foundation. https://www.bhf.org.uk/informationsupport/heart-matters-magazine/activity/exercises-heart-health#:~:text=How%20much%20aerobic%20exercise%20a,next%20 day%20before%20doing%20more.

[CR5] BHF (2022) BHF analysis of ONS Nomis (England & Wales), NRS (Scotland) and NISRA: mortality data

[CR6] BHF (2016) In: The National Audit of Cardiac Rehabilitation: Annual Statistical Report

[CR7] Birckhead B, Khalil C, Liu X, Conovitz S, Rizzo A, Danovitch I, Bullock K, Spiegel B (2019) Recommendations for methodology of virtual reality clinical trials in health care by an international working group: iterative study. JMIR Ment Health 6(1):e11973. 10.2196/11973 30702436 PMC6374734

[CR8] Blazer DG, Hybels CF (2010) Shortness of breath as a predictor of depressive symptoms in a community sample of older adults. Int J Geriatr Psychiatry 25(10):1080–1084. 10.1002/gps.2477 20872930 PMC3039879

[CR9] Boeldt D, McMahon E, McFaul M, Greenleaf W (2019) Using virtual reality exposure therapy to enhance treatment of anxiety disorders: identifying areas of clinical adoption and potential obstacles. Front Psychiatry 10:773. 10.3389/fpsyt.2019.00773 31708821 PMC6823515

[CR10] Bozkurt B, Fonarow GC, Goldberg LR, Guglin M, Josephson RA, Forman DE, Lin G, Lindenfeld J, O’Connor C, Panjrath G, Piña IL, Shah T, Sinha SS, Wolfel E (2021) Cardiac rehabilitation for patients with heart failure: JACC expert panel. J Am Coll Cardiol 77(11):1454–1469. 10.1016/j.jacc.2021.01.030 33736829

[CR11] Bruce RM, Jolley C, White MJ (2019) Control of exercise hyperpnoea: contributions from thin-fibre skeletal muscle afferents. Exp Physiol 104(11):1605–1621. 10.1113/EP087649 31429500

[CR12] Bruce RM, Rafferty GF, Finnegan SL, Sergeant M, Pattinson KTS, Runswick OR (2025) Incongruent virtual reality cycling exercise demonstrates a role of perceived effort in cardiovascular control. J Physiol. 10.1113/JP287421 PMC12456392 39754534

[CR13] Buhle J, Wager TD (2010) Performance-dependent inhibition of pain by an executive working memory task. Pain 149(1):19–26. 10.1016/j.pain.2009.10.027 20129735 PMC4229048

[CR14] Çakal B, Yıldırım M, Emren SV (2022) Kinesiophobia, physical performance, and health-related quality of life in patients with coronary artery disease. Postepy Kardiol Interwencyjnej 18(3):246–254. 10.5114/aic.2022.122892 36751297 PMC9885221

[CR15] Czub MJ, P (2021) Exercise in virtual reality with a muscular avatar influences performance on a weightlifting exercise. J Psychosocial Res Cyberspace 15. 10.5817/CP2021-3-10

[CR16] Dibben GO, Faulkner J, Oldridge N, Rees K, Thompson DR, Zwisler AD, Taylor RS (2023) Exercise-based cardiac rehabilitation for coronary heart disease: a meta-analysis. Eur Heart J 44(6):452–469. 10.1093/eurheartj/ehac747 36746187 PMC9902155

[CR17] Farris SG, Abrantes AM, Bond DS, Stabile LM, Wu WC (2019) Anxiety and fear of exercise in cardiopulmonary rehabilitation: patient and practitioner perspectives. J Cardiopulm Rehabil Prev 39(2):E9–e13. 10.1097/hcr.0000000000000401 30801438 PMC6391737

[CR18] Finnegan SL, Dearlove DJ, Morris P, Freeman D, Sergeant M, Taylor S, Pattinson KTS (2023) Breathlessness in a virtual world: an experimental paradigm testing how discrepancy between VR visual gradients and pedal resistance during stationary cycling affects breathlessness perception. PLoS ONE 18(4):e0270721. 10.1371/journal.pone.0270721 37083693 PMC10120935

[CR19] Freeman D, Haselton P, Freeman J, Spanlang B, Kishore S, Albery E, Denne M, Brown P, Slater M, Nickless A (2018) Automated psychological therapy using immersive virtual reality for treatment of fear of heights: a single-blind, parallel-group, randomised controlled trial. Lancet Psychiatry 5(8):625–632. 10.1016/S2215-0366(18)30226-8 30007519 PMC6063994

[CR20] Frey DP, Bauer ME, Bell CL, Low LK, Hassett AL, Cassidy RB, Boyer KD, Sharar SR (2019) Virtual reality analgesia in labor: the VRAIL pilot study—a preliminary randomized controlled trial suggesting benefit of immersive virtual reality analgesia in unmedicated laboring women. Anesth Analg 128(6):e93–e96. 10.1213/ANE.0000000000003649 31094789

[CR21] García-Bravo S, Cuesta-Gómez A, Campuzano-Ruiz R, López-Navas MJ, Domínguez-Paniagua J, Araújo-Narváez A (2021) Cano-de-la-Cuerda, R. Virtual reality and video games in cardiac rehabilitation programs. A systematic review. Disability Rehabilit 43(4):448–457. 10.1080/09638288.2019.1631892 31258015

[CR22] Goddard M (2017) The EU General Data Protection Regulation (GDPR): European regulation that has a global impact. Int J Market Res 59(6):703–705

[CR23] Hanania NA, O’Donnell DE (2019) Activity-related dyspnea in chronic obstructive pulmonary disease: physical and psychological consequences, unmet needs, and future directions. Int J Chron Obstruct Pulmon Dis 14:1127–1138. 10.2147/COPD.S188141 31213793 PMC6538882

[CR24] Hayen A, Herigstad M, Pattinson KT (2013) Understanding dyspnea as a complex individual experience. Maturitas 76(1):45–50. 10.1016/j.maturitas.2013.06.005 23849705

[CR25] Inbar O, Oren A, Scheinowitz M, Rotstein A, Dlin R, Casaburi R (1994) Normal cardiopulmonary responses during incremental exercise in 20–70-yr-old men. Med Sci Sports Exerc 26(5):538–546 8007799

[CR26] Jiang L, Wang Y, Zhang Y, Li R, Wu H, Li C, Wu Y, Tao Q (2019) The reliability and validity of the center for epidemiologic studies depression scale (CES-D) for Chinese University students. Front Psychiatry 10:315. 10.3389/fpsyt.2019.00315 31178764 PMC6537885

[CR27] Johnson MJ, Yorke J, Hansen-Flaschen J, Lansing R, Ekstrom M, Similowski T, Currow DC (2017) Towards an expert consensus to delineate a clinical syndrome of chronic breathlessness. Eur Respir J 49(5). 10.1183/13993003.02277-2016 28546269

[CR28] Kayikcioglu O, Bilgin S, Seymenoglu G, Deveci A (2017) State and trait anxiety scores of patients receiving intravitreal injections. Biomed Hub 2(2):1–5. 10.1159/000478993 PMC6945947 31988910

[CR29] Evenson KR, Ridenour TA, Bagwell J, et al. Sustaining Physical Activity Following Cardiac Rehabilitation Discharge [Internet]. Research Triangle Park (NC): RTI Press; 2021 Feb. 10.3768/rtipress.2021.rr.0043.2102 34033307

[CR30] Kennedy RS, Lane NE, Berbaum KS, Lilienthal MG (1993) Simulator sickness questionnaire: an enhanced method for quantifying simulator sickness. Int J Aviat Psychol 3:203–220

[CR31] Klompstra LV, Jaarsma T, Strömberg A (2014) Exergaming in older adults: a scoping review and implementation potential for patients with heart failure. Eur J Cardiovasc Nurs 13(5):388–398. 10.1177/1474515113512203 24198306 PMC4361694

[CR32] Kouijzer M, Kip H, Bouman YHA, Kelders SM (2023) Implementation of virtual reality in healthcare: a scoping review on the implementation process of virtual reality in various healthcare settings. Implement Sci Commun 4(1):67. 10.1186/s43058-023-00442-2 37328858 PMC10276472

[CR33] Kroenke K, Spitzer RL, Williams JB (2001) The PHQ-9: validity of a brief depression severity measure. J Gen Intern Med 16(9):606–613. 10.1046/j.1525-1497.2001.016009606.x 11556941 PMC1495268

[CR34] Krupp LB, LaRocca NG, Muir-Nash J, Steinberg AD (1989a) The fatigue severity scale. Application to patients with multiple sclerosis and systemic lupus erythematosus. Arch Neurol 46(10):1121–1123. 10.1001/archneur.1989.00520460115022 2803071

[CR35] Krupp LB, LaRocca NG, Muir-Nash J, Steinberg AD (1989b) The fatigue severity scale: application to patients with multiple sclerosis and systemic lupus erythematosus. Arch Neurol 3:203–220 10.1001/archneur.1989.00520460115022 2803071

[CR36] Kyaw BM, Saxena N, Posadzki P, Vseteckova J, Nikolaou CK, George PP, Divakar U, Masiello I, Kononowicz AA, Zary N, Car T, L (2019) Virtual reality for health professions education: systematic review and meta-analysis by the digital health education collaboration. J Med Internet Res 21(1):e12959. 10.2196/12959 30668519 PMC6362387

[CR37] Li A, Montano Z, Chen VJ, Gold JI (2011) Virtual reality and pain management: current trends and future directions. Pain Manag 1(2):147–157. 10.2217/pmt.10.15 21779307 PMC3138477

[CR38] Marlow LL, Faull OK, Finnegan SL, Pattinson KTS (2019) Breathlessness and the brain: the role of expectation. Curr Opin Support Palliat Care 13(3):200–210. 10.1097/spc.0000000000000441 31306187 PMC6686955

[CR39] McCaul KD, Malott JM (1984) Distraction and coping with pain. Psychol Bull 95(3):516–533. https://www.ncbi.nlm.nih.gov/pubmed/6399756 6399756

[CR40] McCracken LM (1997) Attention to pain in persons with chronic pain: a behavioral approach. Behav ther 28:271–284. 10.1016/S0005-7894(97)80047-0

[CR41] McMahon SR, Ades PA, Thompson PD (2017) The role of cardiac rehabilitation in patients with heart disease. Trends Cardiovasc Med 27(6):420–425. 10.1016/j.tcm.2017.02.005 28318815 PMC5643011

[CR42] Mehling WE, Acree M, Stewart A, Silas J, Jones A (2018) The multidimensional assessment of interoceptive awareness, version 2 (MAIA-2). PLoS ONE 13(12):e0208034. 10.1371/journal.pone.0208034 30513087 PMC6279042

[CR43] Moore SM, Charvat JM, Gordon NH, Pashkow F, Ribisl P, Roberts BL, Rocco M (2006) Effects of a change intervention to increase exercise maintenance following cardiac events. Ann Behav Med 31(1):53–62. 10.1207/s15324796abm3101_9 16472039

[CR44] Mouatt B, Smith A, Mellow M, Parfitt G, Smith R, Stanton T (2020) The use of virtual reality to influence motivation, affect, enjoyment, and engagement during exercise: a scoping review. Virtual Reality 1. 10.3389/frvir.2020.564664

[CR45] Mytinger M, Nelson RK, Zuhl M (2020) Exercise prescription guidelines for cardiovascular disease patients in the absence of a baseline stress test. J Cardiovasc Dev Dis 7(2). 10.3390/jcdd7020015 PMC7344739 32349219

[CR46] Ng Y-LM, Ho F, Ip FK, Fu P, K (2019) Effectiveness of virtual and augmented reality-enhanced exercise on physical activity, psychological outcomes, and physical performance: a systematic review and meta-analysis of randomized controlled trials. Computers Hum Behav 99:278–291

[CR47] NHS (2024) NICE Talking Therapies Guidance. NHS , page 46 https://england.nhs.uk/wp-content/uploads/2018/06/nhs-talking-therapies-manual-v7.1-updated.pdf

[CR48] NICE (2018) Resource Impact Report: Chronic Obstructive Pulmonary Disease in Over 16s: Diagnosis and Management (update) (NG115). https://www.nice.org.uk/guidance/ng115/resources/resource-impact-report-pdf-6602803741

[CR49] NIHR (2024) Briefing Notes for Researchers: Public involvment in NHS, health and social care research. https://www.nihr.ac.uk/briefing-notes-researchers-public-involvement-nhs-health-and-social-care-research

[CR50] O’Donnell DE, Milne KM, James MD, de Torres JP, Neder JA (2020) Dyspnea in COPD: new mechanistic insights and management implications. Adv Ther 37(1):41–60. 10.1007/s12325-019-01128-9 31673990 PMC6979461

[CR51] Okwose NC, O’Brien N, Charman S, Cassidy S, Brodie D, Bailey K, MacGowan GA, Jakovljevic DG, Avery L (2020) Overcoming barriers to engagement and adherence to a home-based physical activity intervention for patients with heart failure: a qualitative focus group study. BMJ Open 10(9):e036382. 10.1136/bmjopen-2019-036382 PMC7507843 32958484

[CR52] Park MJ, Kim DJ, Lee U, Na EJ, Jeon HJ (2019) A literature overview of virtual reality (VR) in treatment of psychiatric disorders: recent advances and limitations. Front Psychiatry 10:505. 10.3389/fpsyt.2019.00505 31379623 PMC6659125

[CR53] Polkey MI, Moxham J (2006) Attacking the disease spiral in chronic obstructive pulmonary disease. Clin Med (Lond) 6(2):190–196. 10.7861/clinmedicine.6-2-190 16688981 PMC4953207

[CR54] Radloff, L. S. (1977). The CES-D Scale: A self-report depression scale for research in the general population. Applied Psychological Measurement, 1(3), 385–401. 10.1177/014662167700100306

[CR55] Radloff LS (1991) The use of the center for epidemiologic studies depression scale in adolescents and young adults. J Youth Adolesc 20(2):149–166. 10.1007/BF01537606 24265004

[CR56] Reiss S, Peterson RA, Gursky DM, McNally RJ (1986) Anxiety sensitivity, anxiety frequency and the prediction of fearfulness. Behav Res Ther 24:1–8. 10.1016/0005-7967(86)90143-9 3947307

[CR57] Resurrección DM, Motrico E, Rubio-Valera M, Mora-Pardo JA, Moreno-Peral P (2018) Reasons for dropout from cardiac rehabilitation programs in women: a qualitative study. PLoS ONE 13(7):e0200636. 10.1371/journal.pone.0200636 30011341 PMC6047805

[CR58] Rochester CL, Vogiatzis I, Holland AE, Lareau SC, Marciniuk DD, Puhan MA, Spruit MA, Masefield S, Casaburi R, Clini EM, Crouch R, Garcia-Aymerich J, Garvey C, Goldstein RS, Hill K, Morgan M, Nici L, Pitta F, Ries AL (2015) An official american thoracic society/european respiratory society policy statement: enhancing implementation, use, and delivery of pulmonary rehabilitation. Am J Respir Crit Care Med 192(11):1373–1386. 10.1164/rccm.201510-1966ST 26623686

[CR59] Runswick OS, Rafferty L, Søvdsnes GF, Knudsen H, Sefton L, Taylor S (2023) The effects of congruent and incngruent immersive virtual reality modulated exercise envirnoments in healthy individuals: a pilot study. Int J Hum Comput Interact. 10.1080/10447318.2023.2276524

[CR60] Salkovskis PM, Rimes KA, Warwick HM, Clark DM (2002) The health anxiety inventory: development and validation of scales for the measurement of health anxiety and hypochondriasis. Psychol Med 32(5):843–853. 10.1017/s0033291702005822 12171378

[CR61] Scano G, Gigliotti F, Stendardi L, Gagliardi E (2013) Dyspnea and emotional states in health and disease. Respir Med 107(5):649–655. 10.1016/j.rmed.2012.12.018 23347530

[CR62] Sevinc V, Berkman MI (2020) Psychometric evaluation of simulator sickness questionnaire and its variants as a measure of cybersickness in consumer virtual environments. Appl Ergon 82:102958. 10.1016/j.apergo.2019.102958 31563798

[CR63] Solomon BK, Wilson KG, Henderson PR, Poulin PA, Kowal J, McKim DA (2015) A breathlessness catastrophizing scale for chronic obstructive pulmonary disease. J Psychosom Res 79(1):62–68. 10.1016/j.jpsychores.2014.11.020 25498317

[CR64] Spielberger CD, Gonzalez-Reigosa F, Martinez-Urrutia A, Natalicio LF, Natalicio DS (1971) The state-trait anxiety inventory. Revista Interamericana de Psicologia/Interamerican Journal Psychol 5.

[CR65] Spitzer RL, Kroenke K, Williams JB, Löwe B (2006) A brief measure for assessing generalized anxiety disorder: the GAD-7. Arch Intern Med 166(10):1092–1097. 10.1001/archinte.166.10.1092 16717171

[CR66] Sullivan, M. J. L., Bishop, S. R., & Pivik, J. (1995). The Pain Catastrophizing Scale: Development and validation. Psychological Assessment, 7(4), 524–532. 10.1037/1040-3590.7.4.524

[CR67] Taylor RR, Jason LA, Torres A (2000) Fatigue rating scales: an empirical comparison. Psychol Med 30(4):849–856. 10.1017/s0033291799002500 11037093

[CR68] Taylor RS, Dalal HM, McDonagh STJ (2022) The role of cardiac rehabilitation in improving cardiovascular outcomes. Nat Rev Cardiol 19(3):180–194. 10.1038/s41569-021-00611-7 34531576 PMC8445013

[CR69] Turk-Adawi KI, Grace SL (2015) Narrative review comparing the benefits of and participation in cardiac rehabilitation in high-, middle- and low-income countries. Heart Lung Circ 24(5):510–520. 10.1016/j.hlc.2014.11.013 25534902 PMC4527841

[CR70] Van den Bergh O, Witthoft M, Petersen S, Brown RJ (2017) Symptoms and the body: taking the inferential leap. Neurosci Biobehav Rev 74:185–203. 10.1016/j.neubiorev.2017.01.015 28108416

[CR71] Watson D, Clark LA, Tellegen A (1988) Development and validation of brief measures of positive and negative affect: the PANAS scales. J Pers Soc Psychol 54(6):1063–1070. 10.1037//0022-3514.54.6.1063 3397865

[CR72] Weber S, Weibel D, Mast FW (2021) How to Get there when you are there already? Defining presence in virtual reality and the importance of perceived realism. Front Psychol 12:628298. 10.3389/fpsyg.2021.628298 34025504 PMC8136250

[CR73] Weech S, Kenny S, Barnett-Cowan M (2019) Presence and cybersickness in virtual reality are negatively related: a review. Front Psychol 10. 10.3389/fpsyg.2019.00158 PMC6369189 30778320

[CR74] Weibel D, Wissmath B, Mast FW (2010) Immersion in mediated environments: the role of personality traits. Cyberpsychology Behav Social Netw 13(3):251–256. 10.1089/cyber.2009.0171 20557243

[CR75] Wilkinson M, a. J. SB F (2021) A mini review of presence and immersion in virtual reality. Hum Factors Ergon Soc 65(1)

[CR76] Witmer BG, Singer MJ (1998) Measuring presence in virtual environments: a presence questionnaire. Presence 7:225–240

[CR77] Xiang X, Huang L, Fang Y, Cai S, Zhang M (2022) Physical activity and chronic obstructive pulmonary disease: a scoping review. BMC Pulm Med 22(1):301. 10.1186/s12890-022-02099-4 35932050 PMC9354440

[CR78] Yorke J, Swigris J, Russell AM, Moosavi SH, Man Kwong N, Longshaw G, M., Jones PW (2011) Dyspnea-12 is a valid and reliable measure of breathlessness in patients with interstitial lung disease. Chest 139(1):159–164. 10.1378/chest.10-0693 20595454 PMC3035488

